# Electrophysiological hallmarks for event relations and event roles in working memory

**DOI:** 10.3389/fnins.2023.1282869

**Published:** 2024-01-24

**Authors:** Xinchi Yu, Jialu Li, Hao Zhu, Xing Tian, Ellen Lau

**Affiliations:** ^1^Program of Neuroscience and Cognitive Science, University of Maryland, College Park, MD, United States; ^2^Department of Linguistics, University of Maryland, College Park, MD, United States; ^3^Division of Arts and Sciences, New York University Shanghai, Shanghai, China; ^4^Shanghai Key Laboratory of Brain Functional Genomics (Ministry of Education), School of Psychology and Cognitive Science, East China Normal University, Shanghai, China; ^5^NYU-ECNU Institute of Brain and Cognitive Science at NYU Shanghai, Shanghai, China

**Keywords:** event role, relation, working memory, pinging, object files, object indexicals

## Abstract

The ability to maintain events (i.e., interactions between/among objects) in working memory is crucial for our everyday cognition, yet the format of this representation is poorly understood. The current ERP study was designed to answer two questions: How is maintaining events (e.g., the tiger hit the lion) neurally different from maintaining item coordinations (e.g., the tiger and the lion)? That is, how is the event relation (present in events but not coordinations) represented? And how is the agent, or initiator of the event encoded differently from the patient, or receiver of the event during maintenance? We used a novel picture-sentence match-across-delay approach in which the working memory representation was “pinged” during the delay, replicated across two ERP experiments with Chinese and English materials. We found that maintenance of events elicited a long-lasting late sustained difference in posterior-occipital electrodes relative to non-events. This effect resembled the negative slow wave reported in previous studies of working memory, suggesting that the maintenance of events in working memory may impose a higher cost compared to coordinations. Although we did not observe significant ERP differences associated with pinging the agent vs. the patient during the delay, we did find that the ping appeared to dampen the ongoing sustained difference, suggesting a shift from sustained activity to activity silent mechanisms. These results suggest a new method by which ERPs can be used to elucidate the format of neural representation for events in working memory.

## 1 Introduction

The ability to represent events – not only single objects, but also the interactions between/among them – in working memory is crucial for our everyday cognition. It is clear that we can maintain *ad hoc* events in working memory: for example, if we see a lion hit an elephant, we are able to run subsequent mental computations that make use of the relations that compose the event representation, even after the hitting itself is over (e.g., inferring that the elephant is in pain or answering a question about who was hit). However, the implementational *format* of events in working memory remains elusive. In the current study, we introduce an ERP (event-related potential) picture – phrase matching paradigm that provides a novel measure of event representation in working memory.

In the following introduction, we first provide an overview of the “neural object indexical theory” (Section 1.1), a theoretical framework for the working memory representation of multiple objects which forms the background for subsequent questions about the neural representation of event relations between objects. We also review previous research on the negative slow wave (NSW), an ERP marker for the number of indexicals in working memory which will be one of the primary components of interest in our current experiment. Then we overview the existing bodies of literature on the neural representation of event relations and event roles (Section 1.2), which together support the research questions of the current paper.

### 1.1 The neural object indexical theory

#### 1.1.1 Evidence for the neural object indexical theory

The question of how event relations between objects are represented “on the fly” has become more tractable after major advances in the last 30 years in understanding how individual objects are represented and related to the local spatial context in working memory. This line of work suggests that the representation of multiple objects in working memory is supported by a limited set of indexicals or pointers also known as object files (Pylyshyn, 1989, 2001; Kahneman et al., 1992; Xu and Chun, 2007; Carey, 2009; Xie and Zhang, 2017; Brody, 2020; Zhu et al., 2020; Quilty-Dunn and Green, 2021; Quilty-Dunn et al., 2022; Thyer et al., 2022; for a review see Yu and Lau, 2023). The number of discrete object indexicals is limited, classically suggested to have a limit of around 4 (Luck and Vogel, 1997, 2013; Cowan, 2001; Awh et al., 2007; Zhang and Luck, 2008; Xu and Chun, 2009; Ngiam et al., 2022). Various features can be attached to these indexicals, enabling us to tell apart objects with some of the same features, and to distinguish the existence of multiple objects even when their features are identical other than spatial location (Leslie et al., 1998; Treisman, 1998). For example, if two otherwise identical flowers appear at different locations in one’s visual field, typically one is still able to distinguish this from a one-flower situation and to continue to represent the existence of both flowers in the scene, even when direct visual information is interrupted by an occluding screen. This can be understood by positing the maintenance of two object indexicals in working memory, each attaching to its corresponding set of features (in this example, the two sets of features are the same except for location). In contrast, neuropsychological patients with the disorder of simultanagnosia struggle at representing two objects but not one (Coslett and Saffran, 1991; Friedman-Hill et al., 1995; Rafal, 2001), suggesting deficits related to maintaining these object indexicals.

**FIGURE 1:**
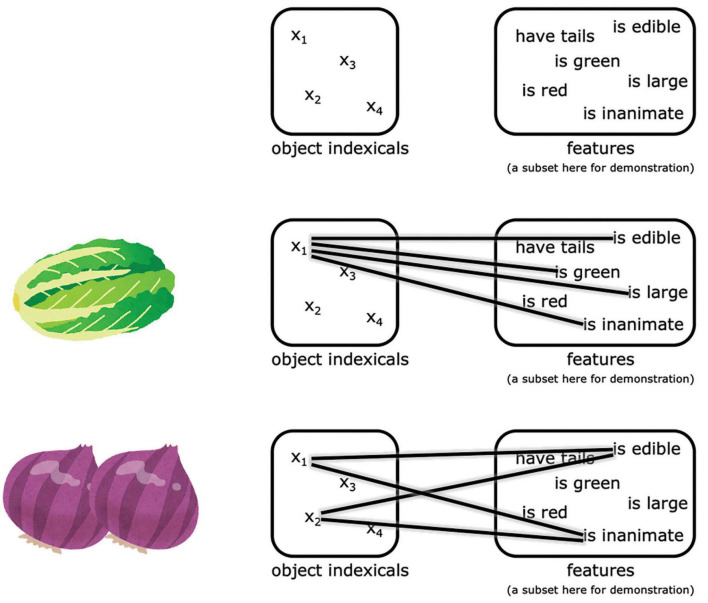
An illustration of the representations proposed by neural object indexical theory. The object indexicals are hosted in some subregions of the posterior parietal cortex, while the features are represented in other regions. The pictures of the Napa cabbage and onions were adapted from IRASUTOYA (https://www.irasutoya.com/), which is free for non-commercial use.

Neuroimaging studies have suggested that these object indexicals are hosted in subregions of the posterior parietal cortex, what we will term here the neural object indexical theory (Xu and Chun, 2007, 2009; Balaban et al., 2019). This proposal is based on the observation that activity in some subregions of the posterior parietal cortex reach a plateau beyond 3–4 items (which is the classical working memory capacity), as measured by fMRI (Todd and Marois, 2004; Song and Jiang, 2006; Xu and Chun, 2006; Mitchell and Cusack, 2008; Matsuyoshi et al., 2010; Robitaille et al., 2010; Knops et al., 2014) and MEG (Robitaille et al., 2010). Consistent with the object indexical theory, evidence from neural studies suggest that these posterior parietal representations do not encode object features themselves (Xu and Chun, 2006; Naughtin et al., 2016); rather, these indexicals connect to features represented in other regions, as illustrated in Figure 1^1^ (Xu, 2007; Naughtin et al., 2016). This theory is also consistent with the proposal that the posterior parietal cortex is crucial to working memory in a more general sense (Bettencourt and Xu, 2016; Jia et al., 2021; for a review see Xu, 2017).

Most visual working memory researchers today assume representations that function like object indexicals, because extreme versions of object-based-all-or-none or feature-only theories for visual working memory cannot account for the behavioral phenomena (for a review see Ngiam, 2023). However, lively debate continues about whether the number of these indexicals is fixed or flexible based on the amount of content that needs to be encoded on each (Brady and Alvarez, 2015; Ngiam et al., 2019), as well as the representational format of features, e.g., whether the features are represented separately or (at least some of them) can be represented conjunctively (Swan and Wyble, 2014; Matthey et al., 2015; Schneegans and Bays, 2017; Hedayati et al., 2022; see Schneegans and Bays, 2019 for a review). While these questions are important, for the purposes of our current investigation of the encoding of event relations between object indexicals, the important shared assumption across all these frameworks is just that increasing the number of indexicals to be represented results in a corresponding increase in processing cost across the maintenance period.

#### 1.1.2 Negative slow wave (NSW): a potential indicator for the object indexical system in working memory

Our methodology for investigating events will center around an ERP component called the negative slow wave (NSW), which is thought to reflect working memory representations related to the object indexical system. Many previous ERP studies of visual working memory have observed sustained negative differences tracking working memory load for multiple objects by using a visual hemifield design that yields a response known as the contralateral delay activity (CDA) (Vogel and Machizawa, 2004). In CDA studies, multiple visual objects are presented on both sides of the visual field, but participants are asked to attend to only one side on any given trial. By hypothesis, ERPs from electrodes contralateral to the attended side will more strongly reflect the working memory representation, so corresponding posterior-occipital electrodes from each side of the scalp are subtracted from each other to compute the contralateral delay activity measure. Many studies have shown that the amplitude of the resulting CDA shows a sustained negativity that is systematically increased with an increasing number of objects to be maintained, up to the individual capacity limit of 3–4 (for an extensive review see Luria et al., 2016). Therefore the CDA has been considered as a potential hallmark for the object indexical system (e.g., Gao et al., 2013; Hakim et al., 2019; Quilty-Dunn and Green, 2021; Cai et al., 2022).

Because our current study was designed to focus on conceptual rather than visuo-spatial representation of events (i.e., the representation shared across linguistic and visual processes), we cannot expect a systematic relationship between working memory representation and visual hemifield. However, another line of visual working memory studies has demonstrated that modulation of the sustained ERP amplitude by number of maintained items can be observed even without manipulating visual hemifield. Since the measure in these studies does not depend on contralateral-ipsilateral subtraction, the response is called instead the negative slow wave. The NSW has been observed in posterior-occipital electrodes with a similar time course to the CDA (Ruchkin et al., 1992; Klaver et al., 1999; Fukuda et al., 2015; Diaz et al., 2021; for a review see Feldmann-Wüstefeld, 2021).^2^ In visual delay-match-to-sample experiments where the sample objects were presented centrally or bilaterally, the NSW has been found to increase in amplitude (i.e., become more negative) as the number of items to be retained increases (Ruchkin et al., 1992; Mecklinger and Pfeifer, 1996; Klaver et al., 1999; Fukuda et al., 2015; Diaz et al., 2021; Feldmann-Wüstefeld, 2021), and reaches a plateau beyond 3–4 items (Fukuda et al., 2015; Zhou and Thomas, 2015; Feldmann-Wüstefeld, 2021), just like the CDA.^3^ Therefore, here we use the magnitude of the NSW as an index of working memory load related to the object indexical system.

### 1.2 What’s different about events? Event relations and event roles

What needs to be represented when representing events involving two objects (i.e., participants, entities; for example, “the lion hit the elephant”) in working memory, compared to simply representing two individual objects together (e.g., “the lion and the elephant”)? One piece is simply representing that the two individuals are participating in a particular event of hitting, what we will term here “event relations.” The other piece is representing the particular roles that the individuals are playing in the event (i.e., agents and patients), which we will term here “event roles.” In the current article, our major interest lies in these *conceptual* representations that are shared across visual and linguistic processes (cf. Jackendoff, 1987; Wurm and Caramazza, 2019; Fitch, 2020; Mahon and Kemmerer, 2020). That is, we are interested in the representation that is shared both when one is viewing the scene where a lion hits an elephant, and when one reads a sentence like “the lion hit the elephant.”

#### 1.2.1 The neural representation of event relations

**FIGURE 2:**
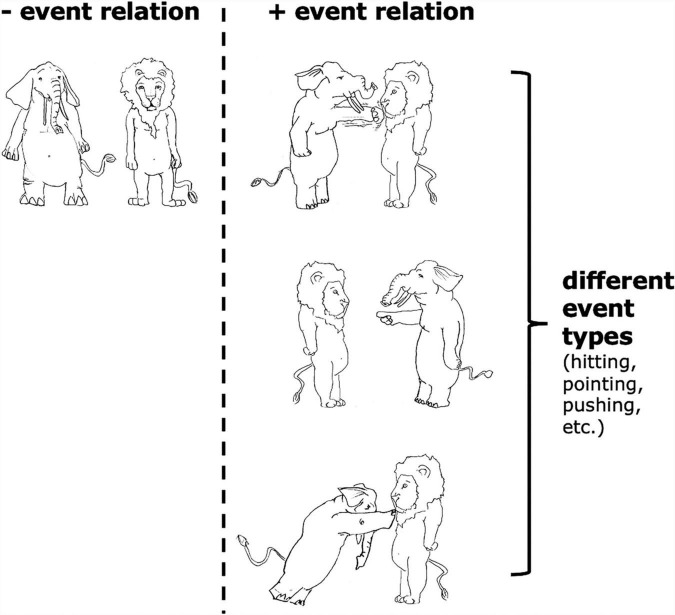
Examples of situations with and without “event relations,” as well as examples of different “event types.”

By event relation, we refer to an intuitive distinction between situations where an elephant hits/points at/pushes a lion vs. an elephant and a lion simply standing beside each other and not doing anything to each other (see Figure 2). The difference between these two kinds of situations is whether there is an event relating the two objects. Of course, there are also different *types* of event relations (i.e., event types), e.g., hitting, pointing at, pushing; but the focus of the current article is how our neural system responds to the existence of an event relation at all in working memory, and not how we represent different event types.

**Inferring event relations**. Event relations can be inferred from both visual and linguistic stimuli. For example, we can use visual information to determine whether there is an event relation for each situation in Figure 2; we can also use linguistic cues to determine whether an event relation is described by phrases like “the lion and the elephant” vs. “the lion hit the elephant.”

Although many visual perception studies have addressed the speed of extracting the event type from a visual image (Dobel et al., 2007b; Hafri et al., 2013; de la Rosa et al., 2014), less is known about how fast the brain detects whether the image depicts a situation including an event relation at all; the only study we are aware of is Hultén et al. (2014). Using MEG, Hultén and colleagues observed that visual events (e.g., where a lion hits an elephant), compared to visual coordinations (e.g., where a lion and an elephant were simply standing beside each other), triggered an early (∼ 200 ms) difference in left occipital regions. However, based on its location (occipital regions) the effect observed in Hultén et al. (2014) is likely visual rather than conceptual in nature.

Similarly, although many neural studies of language have investigated the representation of event types and event argument structures, examining the contrast between phrases that describe events and those that don’t has been less common. Gaston (2020, Experiment 4) investigated the difference between linguistic stimuli including event relations compared to coordination using MEG. Gaston examined the brain responses to serially presented, nonsensical coordinate phrases vs. verb phrases (e.g., the | toasty | tractors | and | the | scenic | cathedrals *vs.* the | toasty | tractors | entered | the | scenic | cathedrals) and observed a difference from ∼150 ms to ∼550 ms after the onset of “and” vs. the verb in regions including the transverse temporal sulcus, pSTS (posterior superior temporal sulcus), STS, and ATL (anterior temporal lobe). This effect is in line with the observation of Matchin et al. (2019a), that the presentation of a verb within a sentence elicits a difference starting from ∼450 ms upon onset in the angular gyrus, compared to a baseline consisting of phrases without a verb. Such transient effects may reflect a difference in linguistic structure building and/or the representation of event relations at the conceptual level. We should note, however, that the analysis pipelines of Matchin et al. (2019a) and Gaston (2020) may not be sensitive to long-lasting sustained effects.

**The maintenance of event relations** in working memory. To our knowledge, only two behavioral studies have directly addressed this question (Clevenger and Hummel, 2014; Shen et al., 2021). The results of Clevenger and Hummel (2014) suggested that more relations impose higher working memory load, and the results of Shen et al. (2021) suggested that only two event relations can be maintained in working memory. However, these results remain inconclusive in terms of the *format* of the representation of event relations in working memory; specifically, how are event relations represented with respect to the object indexical system?

One way to approach this question, and the one we pursue in the current study, is to compare the maintenance across a delay period of events like “the tiger hit the elephant” and the maintenance of non-event coordinations like “the tiger and the elephant.” To our knowledge, the MEG study from Hultén et al. (2014) is the only previous neurophysiological study to have examined the maintenance of events compared to coordinations.^4^ In their study, pictures of events and coordinations were presented for 1,500 ms, followed by a speech production task. A numerical difference was observed between events and coordination conditions in a somewhat late time window (from ∼300 ms to at least 1 s) time-locked to picture onset in the posterior parietal cortex. Although this difference did not survive statistical significance in their analysis, this could be due to the small cohort of subjects (*N* = 10).

**FIGURE 3:**
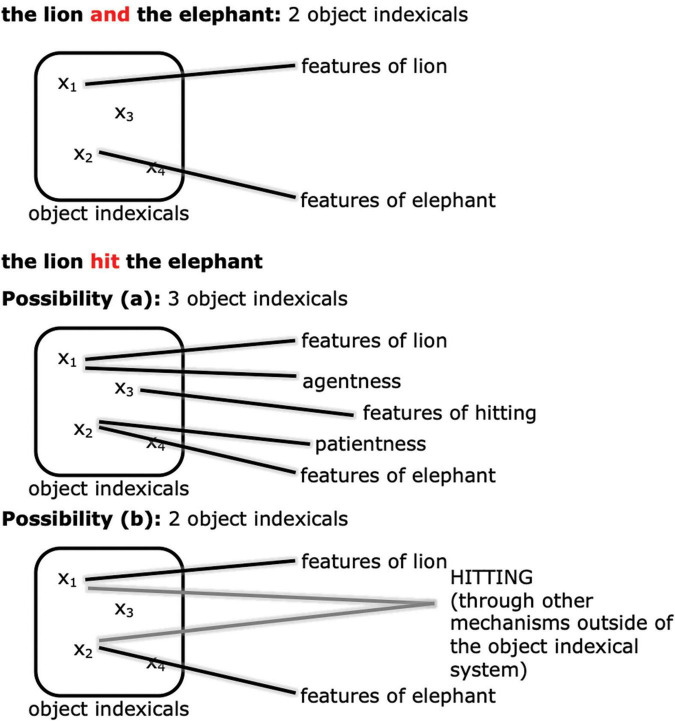
An illustration of the two possibilities of how event relations are represented. Possibility a: the event relation is encoded by another object indexical. Possibility b: the event relation is represented by other mechanisms in, e.g., other regions of the posterior parietal cortex or the hippocampus.

How should we expect event representation in working memory to manifest differently from simply representing a pair of objects? Through the lens of the object indexical system, we think that there are at least two major possibilities (see Figure 3). One possibility (Figure 3a) is that the event relation may be represented with a separate object indexical from the participants (cf. Clevenger and Hummel, 2014; Shen et al., 2021). In other words, adding an event relation increases working memory cost in exactly the same way as adding another object, and counts against the same capacity limit as objects. For example, in the case of “the tiger hit the elephant,” apart from the two object indexicals for the tiger and the elephant, there is a separate object indexical representing the hitting relation, possibly by connecting to the concept HITTING and other features of hitting.^5^ In this case, there will be an NSW-like difference between the maintenance of events and coordinations. Another possibility (Figure 3b) is that the object indexicals in working memory are true to their name and stand in only for objects, not event relations between objects, as implied for example in Altmann and Ekves’ (2019) theory. The relational event encoding might then be accomplished by other posterior parietal circuits, or by other brain regions altogether, such as the hippocampus (e.g., Konkel and Cohen, 2009; Eichenbaum and Cohen, 2014). The object indexical working memory representation might only code such event relations implicitly and indirectly in an object-centered fashion, for example by connecting agent and patient “features” to the object indexicals (for further explanation of agents and patients see Section 1.2.2 below). In this case, there is no reason to predict that there will be an NSW-like difference between the maintenance of events and coordinations.

#### 1.2.2 The neural representation of event roles

Many modern theories assume the existence of generalized roles like agent (the initiator of the event) and patient (the receiver of the event), which characterize the involvement of participants across many different types of events (see Strickland, 2017; Rissman and Majid, 2019; Ünal et al., 2021; Wilson et al., 2022). These roles may be understood to be encoded at the linguistic semantic level or at the non-linguistic conceptual level (see Williams, 2015, 2021 for extensive discussion). How such event roles (also known as “semantic” roles or participant roles) are neurally coded in working memory remains unclear.

**Inferring event roles**. Conceptual event roles can be inferred from visual and linguistic stimuli. In visual behavioral studies (i.e., studies using visual stimuli), it has been observed that the event role of objects can be inferred after only a brief visual presentation (Dobel et al., 2007b; Hafri et al., 2013). In an electroencephalography (EEG) study by Cohn et al. (2017), subjects were presented with comic strips (modified from *the Peanuts*) sequentially, and the ERP responses to frames illustrating the to-be-agent and to-be-patient character were compared. Of the to-be-agent characters, some were making actions suggesting their agentness (“preparatory agents”), while some were not (“non-preparatory agents”). Relative to the patient condition and the non-preparatory agent condition, the preparatory agent condition elicited a left anterior negativity in 300–600 ms and a slightly leftward fronto-central positivity in 500–900 ms.

One way that languages can convey event role assignments is through linguistic case-marking. For example, in Japanese, the conceptual content of *John gave Mary a book* can be realized as “John-NOM Mary-DAT book-ACC gave,” where -NOM, -DAT, -ACC are nominative, dative and accusative case markers *ga*, *ni*, and *o*. Languages can also convey event roles through linear order as well as other structural cues, as in the English active/passive alternation “The cat chased the dog” and “The dog was chased by the cat,” where linear order of the arguments alternate but the event roles stay constant. Prior fMRI work has reported some success in decoding the binding between event roles and objects from the neural response to these kinds of alternations in English (e.g., Frankland and Greene, 2015, 2020; Wang et al., 2016; Lalisse and Smolensky, 2021). However, given the poor temporal resolution of fMRI, these studies were not aimed at disentangling the extraction and maintenance processes of event roles.

**The maintenance of event roles**. A number of behavioral studies have investigated what factors govern accurate maintenance of event roles across time. At least one study suggests that maintaining more event role-object bindings reduces the accuracy of subsequent memory performance (Jessop and Chang, 2020), although it is unclear whether this effect was driven by an increase in the number of objects held in working memory or an increase in the number of event roles bound to these objects (or both). A subsequent study has shown that representing conflicting event roles (e.g., being the agent in one event but the patient in another event) compromises memory performance (Jessop and Chang, 2022). Peng et al. (2018), through a behavioral experiment using the dense sampling method, suggested that event roles are represented at (or even by) different phases of the alpha band (∼ 10 Hz) rhythm; we discuss this possibility in further detail in the Discussion.

To our knowledge, no prior electrophysiological study has examined the maintenance of event roles in working memory. In fact, it is a practical challenge to disentangle the separate representation of the agent and the patient in working memory, given that they participate in the same event. In the current study we introduce an exploratory paradigm adapted from the “pinging” paradigm used in recent working memory studies (Wolff et al., 2015, 2017, 2019; 2020; Fornaciai and Park, 2020; ten Oever et al., 2020; Huang et al., 2021; Duncan et al., 2023; Fan and Luo, 2023). Wolff et al. (2015) conducted a standard delay-match-to-sample working memory task in which the match in the orientation of two Gabor patches needed to be evaluated across a relatively long delay. During the delay period, a high-contrast visual bull’s-eye-shaped stimulus with no orientation information (i.e., the ping) was presented in an attempt to probe the encoding of the orientation information being maintained in memory, similar to the idea of sonar or echolocation. It was found that the otherwise undecodable memory content at that time (the orientation of the sample Gabor patch in this case) was decodable if the ping was presented. In this study and others that followed, the main idea was to probe different working memory contents during retention with the same impulse perturbation (i.e., the ping).

**FIGURE 4:**
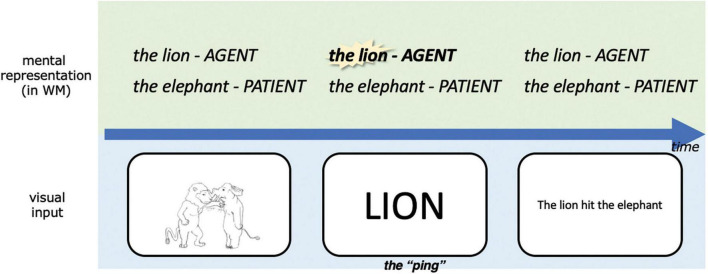
A simplified schematic illustration of our experimental design, highlighting our adapted pinging paradigm. The task was to respond to whether the linguistic expression matched with the picture in this trial upon presentation of the linguistic expression, this was designed to encourage subjects to represent the event *conceptually* during the retention period. WM, working memory. For detailed parameters of the experiment, see Materials and methods.

In the current study, we adapt the pinging paradigm to probe different parts of the same working memory content during retention using different pings (see also Fan et al., 2021). While event representations are being maintained in working memory across the delay period we “ping” the event role with the name of the entity to which it was bound, as illustrated in Figure 4. We ping the agent in half of the event trials and the patient in the other half, which gives us the opportunity to compare the ERP corresponding to an impulse perturbation of agents vs. patients in working memory. Notably, since each animal can be the agent or patient, the agent pings and patient pings are perceptually the same across trials. In order to keep the trial structure consistent throughout the experiment, we also pinged one of the two animals in the coordination condition, even though in this case, absent event roles, both entities have the same status in working memory.

Although little is known thus far about how event roles are neurally encoded, one possibility that we explored in this study is that event roles are represented as a kind of “magnitude”.^6^ Behaviorally, agent-patient event roles have sometimes been observed to interact with spatial position (i.e., left-right), which can be treated as a “magnitude” (e.g., Chatterjee et al., 1995; Maass and Russo, 2003; Dobel et al., 2007a; but see Geminiani et al., 1995; Barrett et al., 2002; Altmann et al., 2006; Kazandjian et al., 2011; Dobel et al., 2014 for conflicting results). Some authors have proposed that regions of parietal cortex represent diverse kinds of scalar and quantity information in a common neural implementation format (Walsh, 2003; Bueti and Walsh, 2009; Summerfield et al., 2020; for a critical review see Martin et al., 2017). One kind of neural evidence taken to support this idea is the finding that magnitudes (including numerosity, magnitude of reward, and magnitude of evidence during decision-making) drive an ERP effect in central-parietal electrodes, that is, the central parietal positivity (CPP, Yeung and Sanfey, 2004; O’Connell et al., 2012; Spitzer et al., 2017; Luyckx et al., 2019; for an overview see O’Connell and Kelly, 2021). For transient (i.e., not constantly-changing) stimuli, the CPP is a late component at around 300 to 700 ms post stimulus-onset (Spitzer et al., 2017; Luyckx et al., 2019), with larger magnitudes being more positive. The hypothesis that event roles are represented as magnitudes would predict an ERP effect with a similar scalp distribution to the CPP.

#### 1.2.3 The current study

**FIGURE 5:**
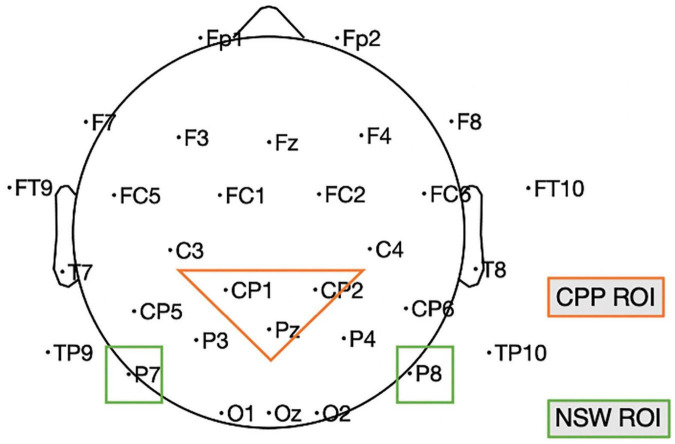
Illustration of the electrodes of our two ROIs in Experiment 1; in Experiment 2 the NSW ROI included Cz, CPz, and Pz because of a different layout. CPP, centro-parietal positivity; NSW, negative slow wave; ROI, region of interest. This is based on the standard 10–20 map provided by the DIPFIT plug-in of EEGLAB.

Our study was designed to investigate neural measures of the maintenance of both event relations and event roles. We ran two experiments with a similar design in two sites, one in Chinese in China (Experiment 1; *N* = 19) and one in English in the US (Experiment 2; *N* = 16). The choice of these two sites/languages was not driven by particular features of the languages, but simply because of practical constraints experienced by the investigators during the COVID-19 pandemic. However, this did allow us to demonstrate that the results extend across two different languages and participant populations. We take posterior-occipital electrodes P7, P8 as the NSW region of interest (ROI) for event relation maintenance, and central-parietal electrodes CP1, CP2, Pz (Experiment 1) and Cz, CPz, Pz (Experiment 2) as the CPP ROI for event role maintenance (see Figure 5).

## 2 Results

### 2.1 Experiment 1

#### 2.1.1 Behavioral results

Across the 19 subjects included in the analyses, the mean proportion of correct responses was 96% (range 89–99%). Mean proportion of correct responses was 93% (when the agent was pinged), 95% (when the patient was pinged) and 98% (in coordination trials). Based on the high accuracy, all trials were analyzed for the EEG analysis.

The mean (± SD) reaction time of correct responses was 1,241 ± 288 ms when the agent was pinged, 1,230 ± 275 ms when the patient was pinged, 1,236 ± 278 ms for all event trials and 903 ± 233 ms for all coordination trials. The reaction times reported here were first averaged within subjects for all the correct trials, then averaged across subjects. Paired *t*-test (two-tailed) indicated no significant difference between the reaction times for agent ping trials and patient ping trials, *t*(18) = 0.6, *p* = 0.56. Paired *t*-test (two-tailed) revealed a significant difference between the reaction times for event trials and coordination trials, *t*(18) = 11.7, *p* < 0.001. It has been observed with the delay-match-to-sample paradigm that the more objects one needs to maintain in working memory, the slower one reacts to the probe stimuli (Hyun et al., 2009; Mitchell and Cusack, 2011; Park et al., 2017). Therefore, the longer reaction time to the probe (in our experiment the linguistic expression) for event trials observed in our current experiment is in line with our interpretation of our EEG results, that the maintenance of events imposes a higher working memory load compared to the maintenance of coordinations, similar to representing an extra object (see Section 3).

#### 2.1.2 EEG results

**FIGURE 6:**
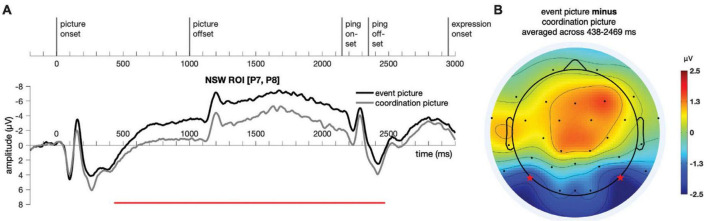
Illustration of the results for epochs time-locked to picture onset in the NSW ROI [electrodes P7 and P8, as marked by the stars in panel **(B)**] for Experiment 1. **(A)** The significant cluster (438–2,469 ms) is marked in red. **(B)** The scalp distribution average across the 438–2,469 ms time window. NSW, negative slow wave; ROI, region of interest.

Event-related potential responses to event pictures and coordination pictures are illustrated in Figure 6. We expected initial evoked responses to the event pictures and coordination pictures to differ because the pictures in the two conditions were physically different across various dimensions, and that responses to the sentences in the two conditions might differ because the form of the correct response differed in the two conditions. Our question of interest was whether we would observe differences across the delay between the picture presentation and the sentence presentation in the posterior electrodes associated with the negative slow wave.

A temporal cluster-based permutation test (Maris and Oostenveld, 2007) was performed on the responses to the event picture and the coordination picture in the time window of −200:3500 ms for the NSW ROI using customized MATLAB scripts. First, potential clusters were identified with point-to-point paired *t*-test at a threshold of alpha = 0.05 (two-tailed). Then, the t values of these potential clusters were compared against a permuted (permutations = 9,999) distribution of t values, with a threshold of alpha = 0.05 (two-tailed). A cluster was identified in the negative slow wave (NSW) ROI, extending from 438 to 2,469 ms after picture onset (*p* = 1 × 10^–4^, Figure 6). The difference manifested as an increased negativity for the event pictures relative to the coordination pictures in these posterior electrodes. Although our analyses focused on the pre-determined NSW ROI, the scalp topography of the cluster suggests that this posterior negativity may have been accompanied by a corresponding (central-)frontal positivity.

**FIGURE 7:**
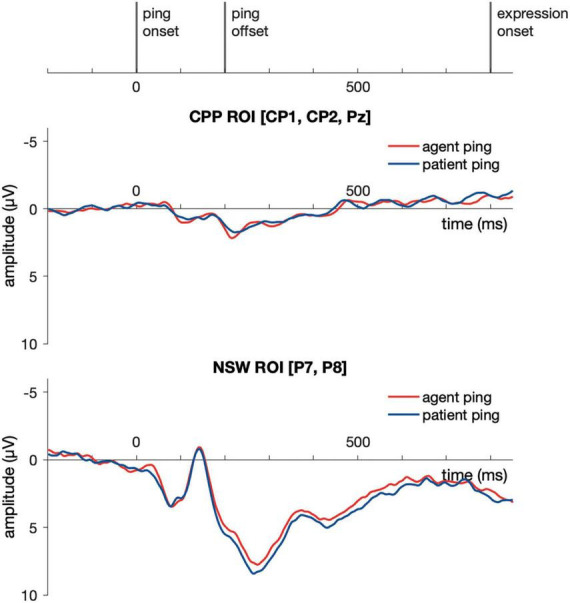
Illustration of the results for epochs time-locked to ping onset in the NSW ROI and the CPP ROI for Experiment 1. NSW, negative slow wave; CPP, centro-parietal positivity; ROI, region of interest.

Event-related potential responses to the delay-period agent ping and the patient ping in the event condition are illustrated in Figure 6. Cluster-based permutation test (Maris and Oostenveld, 2007) was performed comparing the agent ping vs. patient ping conditions in the time window of −200:1800 ms using customized MATLAB scripts using the same approach described above for the event picture comparison. No significant clusters distinguishing the agent ping and patient ping were identified in the CPP ROI, nor in the NSW ROI (Figure 7).

#### 2.1.3 Rationale for experiment 2

In Experiment 1, we observed that maintaining events in working memory, compared to maintaining coordinations, elicited a sustained NSW (negative slow wave) effect. However, during data analysis we discovered an issue in the accuracy of the timing parameters that could have led us to overestimate the duration of the sustained negativity (see Section 4.1.6 for more detail). In Experiment 2, we aimed to replicate the effect in a different population (Experiment 1 was run in Shanghai with stimuli in Chinese and Experiment 2 was run in the US with stimuli in English), with corrected presentation parameters that allow more precise estimation of the duration of the effect.

As visual inspection of the Experiment 1 effects suggested that the presentation of the ping may have acted to reduce or eliminate the sustained negativity, we also took Experiment 2 as an opportunity to evaluate this possibility by presenting the ping somewhat earlier in the trial. We presented the pictures for shorter duration in Experiment 2 (400 ms) compared to Experiment 1 (1,000 ms), as an additional measure to ensure that the sustained effects we observed did indeed reflect delay-period activity rather than continued visual processing.

### 2.2 Experiment 2

#### 2.2.1 Behavioral results

Across the 16 subjects included in the analyses, the mean proportion of correct responses was 92% (range 83–97%). Mean proportion of correct responses was 88% (when the agent was pinged), 91% (when the patient was pinged) and 94% (in coordination trials). Based on the high accuracy, all trials were analyzed for the EEG analysis.

The mean (± SD) reaction time of correct responses was 1,617 ± 345 ms when the agent was pinged, 1,616 ± 357 ms when the patient was pinged, 1,617 ± 345 ms for all event trials and 1,149 ± 349 ms for all coordination trials. The reaction times reported here were first averaged within subjects for all the correct trials, then averaged across subjects. Paired *t*-test (two-tailed) indicated no significant difference between the reaction times for agent ping trials and patient ping trials, *t*(15) = 0.6, *p* = 0.99. Paired *t*-test (two-tailed) revealed a significant difference between the reaction times for event trials and coordination trials, *t*(15) = 9.7, *p* < 0.001, similar to the results in Experiment 1.

#### 2.2.2 EEG results

**FIGURE 8:**
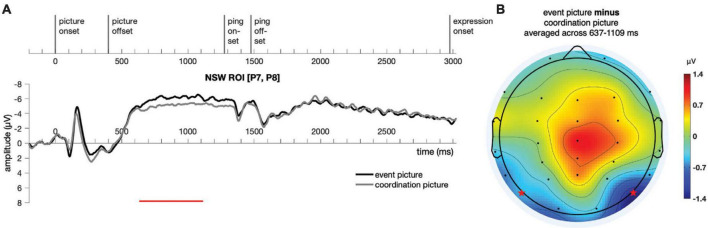
Illustration of the results for epochs time-locked to picture onset in the NSW ROI [electrodes P7 and P8, as marked by the stars in panel **(B)**] for Experiment 2. **(A)** The significant cluster (637–1,109 ms) is marked in red. **(B)** The scalp distribution average across the 637–1,109 ms time window. NSW, negative slow wave; ROI, region of interest.

Similar to Experiment 1, a cluster-based permutation test (Maris and Oostenveld, 2007) was performed on the responses to the event picture and the coordination picture in the time window of −200:3500 ms for the NSW ROI using customized MATLAB scripts. A cluster was identified in the negative slow wave (NSW) ROI, extending from 637 to 1,109 ms after picture onset (*p* = 0.006, Figure 8). As in Experiment 1, the difference manifested as an increased negativity for the event pictures relative to the coordination pictures in these posterior electrodes. We note that the amplitude of the sustained effect was somewhat smaller in Experiment 2 than Experiment 1, perhaps because of the shorter picture duration for encoding in Experiment 2 (see Brady et al., 2016, for a similar correlation between encoding duration and effect size). The scalp topography of this NSW effect was generally similar to the one obtained in Experiment 1. The duration of the sustained effect appeared to extend just up through the onset of the ping screen, after which no significant differences were observed.

**FIGURE 9:**
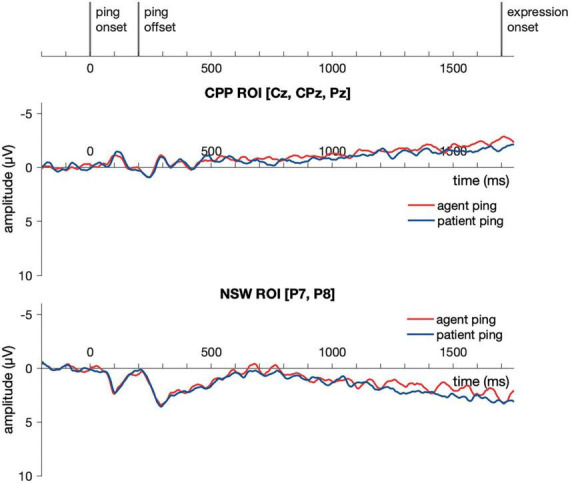
Illustration of the results for epochs time-locked to ping onset (–200:1800 ms) in the NSW ROI and the CPP ROI for Experiment 2. NSW, negative slow wave; CPP, centro-parietal positivity; ROI, region of interest.

Event-related potential responses to the delay-period agent ping and the patient ping in the event condition are illustrated in Figure 9. Similar to Experiment 1, a cluster-based permutation test (Maris and Oostenveld, 2007) was performed comparing the agent ping vs. patient ping conditions in the time window of −200:1800 ms using the same approach described above for the event picture comparison. No significant clusters distinguishing the agent ping and patient ping were identified in the CPP ROI, nor in the NSW ROI.

## 3 Discussion

Our current experiments constitute one of the first electrophysiological studies directly addressing how event relations and event roles (i.e., agent and patient) are maintained in working memory. We report two key findings. First, across Experiments 1 and 2, we saw that the working memory retention of events (e.g., a tiger hit an elephant) relative to simple coordination (e.g., a tiger and an elephant) was hallmarked by a response similar to a negative slow wave (NSW) in posterior-occipital electrodes during the delay period. Maintenance of events resulted in a more negative sustained response compared to coordinations, suggesting higher working memory load based on the standard interpretations of the negative slow wave in prior studies. Our second finding was that in a novel agent vs. patient “pinging” manipulation during the delay, we observed no reliable difference over centro-parietal positivity electrodes or negative slow wave electrodes for agent as compared to patient. Interestingly, however, the sustained negative slow wave effect seemed to be largely dampened after the presentation of the ping, as observed to track the shift of the ping presentation timing from Experiment 1 to Experiment 2.

### 3.1 Representing event relations in working memory

The major difference between the event and coordination conditions was in whether or not an event relation needed to be represented across the delay period. The NSW effect in Experiment 1 onset at ∼ 450 ms and extended even until ∼ 2,500 ms; for Experiment 2, it onset at ∼ 600 ms and extended until ∼ 1,100 ms (approximately when it was interrupted by the ping). This late, *sustained* effect across the two conditions suggests that event relations *can* be represented by sustained activity in working memory. Our current study, in demonstrating a neural measure sensitive to the maintenance of event relations, thus marks a crucial first step toward further elucidating the implementation-level question of *how* event relations are represented in working memory.

What brain regions contributed to the NSW effects in our current study? Although future work with source localization in MEG is needed, we believe that posterior parietal cortex is the most likely candidate. The relatively late onset of the sustained effect here is comparable to the trend toward a sustained effect for event representation in Hultén et al.’s (2014) MEG study, which they localized to posterior parietal cortex. Of course, a posterior parietal source would also be consistent with the previous work reviewed in the introduction emphasizing the contribution of this region to working memory (Robitaille et al., 2010; Fan et al., 2012; Becke et al., 2015; Bettencourt and Xu, 2016; Jia et al., 2021) and event representation (Thompson et al., 2007; Centelles et al., 2011; Wang et al., 2016; Williams et al., 2017; Matchin et al., 2019a,b). Moreover, the posterior parietal cortex has been observed to be involved in both linguistic and visual processing, suggesting that it is hosting conceptual representations (Menenti et al., 2012; Jouen et al., 2015; Baldassano et al., 2017; Zadbood et al., 2017; Liu et al., 2020; Ivanova et al., 2021). Previous work (Summerfield et al., 2020) has proposed that the posterior parietal cortex plays a crucial role in representing “relational structures” (see also O’Reilly et al., 2022), yet the format of this representation remains elusive. One possibility consistent with the negative slow wave (NSW) effect during the maintenance of events vs. coordinations is that the event relation in an event is represented by a separate object indexical apart from the two participants. These “relation indexicals” could be qualitatively different from the indexicals for objects; a possibility that could be tested in future experiments. However, given the absence of localization information in the current study, it is also possible that the NSW we observed was driven by other posterior parietal circuits (or even other brain regions). Further work is needed to disentangle these possibilities.

Interestingly, Lapinskaya et al. (2016) observed a late (from 500 ms to at least 800 ms) parietal-occipital ERP effect between object nouns vs. event nouns and verbs after the presentation of the second word in a two-word similarity judgment task. This effect had a similar scalp distribution to our current effect based on visual inspection. The object nouns were more positive over the posterior electrodes compared to verbs and event nouns, which mirrors the current results in which coordinations elicited a less negative response than events. However, Lapinskaya et al. (2016) used a single-word similarity judgment paradigm, which is quite different than the paradigm of our current study, and did not examine responses over an extended maintenance delay.

We should also note that some behavioral studies have shown that when two objects are involved in an event, participants can actually perform better on working memory tasks than when they are maintaining two separate objects (Stahl and Feigenson, 2014; Ding et al., 2017; Lu et al., 2022; Paparella and Papeo, 2022). This seeming contradiction with our current results remains to be resolved in future research. One possibility is that the number of working memory indexicals used to encode the same input may vary according to task environments, as suggested by studies on visual grouping (Balaban and Luria, 2016; Rabbitt et al., 2017; McCants et al., 2020). Another possibility is that different numbers of indexicals may be used during encoding and maintenance phases, as suggested for visual grouping (Peterson et al., 2015; Thyer et al., 2022).

### 3.2 Representing event roles in working memory

In this study we introduced a novel “pinging” paradigm to attempt to observe evidence of the differential coding of event roles in working memory across the delay. One hypothesis was that event roles are coded similarly to magnitudes, and that this would be associated with differences in the electrodes previously associated with a centro-parietal positivity electrodes suggested to reflect magnitude coding. We failed to find evidence for this hypothesis, as we did not observe a significant effect of the pinged event role over CPP electrodes across the two experiments. This is not definitive evidence against magnitude coding of event roles, as the CPP may not be a general hallmark for all magnitudes. We also note that much of the existing pinging literature has relied on decoding rather than comparing mean differences. For example, Fornaciai and Park (2020), in a numerosity working memory task, identified a significant ERP difference upon sample presentation across conditions, yet failed to identify significant ERP differences upon ping presentation during the retention period across these conditions. In line with prior pinging literature, they did, however, observe that ping presentation was associated with above-chance decodability. Further research is needed to investigate whether ERP amplitudes are generally insensitive to the perturbation responses elicited by pinging.

However, our failure to find agent-patient differences in the ping paradigm may also reflect the fact that magnitude coding is not the format by which event roles are related to object indexicals in working memory. There are clear theoretical challenges for representing event roles as magnitudes. For example, if we consider tools or instruments (e.g., Mary opened the bottle with *the opener*), it is hard to think of this role as being somewhere on a 1-D scale along the same line of agent and patient. This is different from other magnitudes (e.g., numerosity, magnitude of reward, and magnitude of evidence during decision-making), which can be understood as being represented on a 1-D magnitude scale.

What are alternative encoding mechanisms beyond magnitude that could support event role representation? There are currently at least two other major hypotheses about the implementation of event roles. One is that different event roles, as features, are represented by different neural firing patterns. The other is that agent and patient roles are signaled by oscillatory phase. For example, it could be that when an object indexical is representing the agent object, it is most excited at one phase, while when the same object indexical is representing the patient object, it is most excited at another phase. The brain may make use of both representations for different purposes. Some evidence for the first hypothesis comes from an fMRI decoding study of Wang et al. (2016), which successfully decoded (above chance) from the response in a wide range of brain regions whether a certain animal was the agent or the patient in a visual scene. Versions of the second hypothesis have long been discussed in the computational literature (Shastri and Ajjanagadde, 1993; Shastri, 1999, 2002, 2007; Hummel and Holyoak, 2003; Martin and Doumas, 2017), yet have only recently been tested by Peng et al. (2018). Using the dense sampling method for identifying behavioral oscillations, Peng and colleagues found that the object indexicals for agents and patients are most excited at a ∼90 degree lag in the alpha band (∼10 Hz). Results from several other studies are also in line with the possibility that when holding multiple objects (≥ 2) in working memory, different object indexicals are most excited at different oscillatory phases (Jia et al., 2017; Peters et al., 2021; Pomper and Ansorge, 2021; Balestrieri et al., 2022; Chota et al., 2022). Our current results do not provide strong evidence either for or against these hypotheses.

Another dispute over the representation of event roles is whether they are independent or dependent from the event type (see Williams, 2015, 2021). By relation-independent, we refer to abstract roles like agent and patient; by relation-dependent, we refer to action-specific roles such as hitter and hittee, hugger and huggee, etc. On the assumption that roles are relation-dependent, one possibility is that the perception of an independent “agent” role may come from different relation-dependent roles being aligned on some graded dimension of “agentivity” (for example, hitters may be more “agenty” than huggers). While a large body of literature in formal semantics assumes the existence of relation-independent roles (for discussions see Williams, 2021), Dowty (1991) suggested that (proto-)agents and (proto-)patients may rather be characterized by varying numbers of “typical” features, which correspond to graded responses that some prior studies have empirically quantified with subjective ratings (Kako, 2006a,b; Reisinger et al., 2015; Rissman and Lupyan, 2022, for studies with a similar logic using other ratings see Johnson, 1967; Madnani et al., 2010). More research is still needed to resolve this dispute, which in turn will inform whether neural studies should expect to find for consistent “agent” or “patient” representations across event types.

### 3.3 Sustained activity vs. activity-silent mechanisms

Our current results are consistent with the idea that event relations can be maintained in working memory in the form of sustained neural activity, given that we observe a sustained ERP difference between the retention of events and coordinations. Although sustained activity has long been seen as a key mechanism for working memory representation (for reviews see Leavitt et al., 2017; Wang, 2021), at least two lines of research have argued that working memory retention can also sometimes be implemented by “activity-silent mechanisms” (Lewis-Peacock et al., 2012; Stokes, 2015; Rose et al., 2016; Kamiński and Rutishauser, 2020; Beukers et al., 2021) or other mechanisms that do not induce sustained changes in neural metabolism (e.g., Wutz et al., 2020; Lundqvist et al., 2021).

One line of research has utilized visual stimuli, and observed that the amplitude of CDA after presenting two consecutive memory arrays is not always reflective of the load of the first memory array (Berggren and Eimer, 2016; Feldmann-Wüstefeld et al., 2018; Zhang et al., 2021) and that a disruption in sustained activity is not necessarily accompanied by a huge impairment in memory performance (Kreither et al., 2022). Another line of research has utilized linguistic stimuli, and has observed that the maintenance of syntactic dependency is not always accompanied by the presence of the sustained component of SAN (sustained anterior negativity) (McKinnon and Osterhout, 1996; Kaan et al., 2000; Lau and Liao, 2018; Yano and Koizumi, 2018, 2021; Lo and Brennan, 2021; Sprouse et al., 2021; Cruz Heredia et al., 2022; Lau and Yang, 2023; for an earlier review see Lau, 2018).

Our current experiment provides one of the first reports of how event relations are neurally represented across time in working memory. The sustained negative slow wave effect across events and coordinations in Experiment 1 and 2 demonstrated that the working memory maintenance of event relations *can* be realized by sustained activity. However, as Experiment 2 confirmed, this sustained NSW effect was largely dampened by the ping, and yet participants continued to demonstrate high accuracy on the behavioral memory task. This is in line with previous findings in visual working memory studies that an interruption during retention dampened CDA (Berggren and Eimer, 2016; Feldmann-Wüstefeld et al., 2018; Zhang et al., 2021), yet resulted in little detriment to behavioral accuracy (Kreither et al., 2022).^7^ Together, these results suggest that the representational format of the memorandum may shift from sustained activity to activity-silent representation upon interruption. Therefore, our observation that the sustained difference was reduced after the ping suggests that although the working memory maintenance of event relations *can* be realized by sustained activity, it can also be represented in an activity silent format.

### 3.4 Event type and social interactions

In the current study, we focused on how the brain reacts to whether or not there is an event relation at all in working memory, but not how the event type itself (e.g., hitting, kicking) is represented. Previous fMRI studies have converged on the posterior lateral temporal cortex (PLTC, Wurm and Lingnau, 2015; Wurm et al., 2016; Hafri et al., 2017; Walbrin and Koldewyn, 2019; Wurm and Caramazza, 2019) as hosting the representation for event types. Following our interpretation of the negative slow wave effect, it is possible that an indexical for event relations in the PPC (posterior parietal cortex) points to the event type represented in PLTC during working memory maintenance. However, evidence remains mixed on whether the event type representations in PLTC reflect the extraction of event types or the maintenance of event types in working memory, or both (Lu et al., 2016; Zhou et al., 2022).

The NSW effect in our current study could also be interpreted as reflecting social cognition computations in particular, related to the presence of social interaction for events. Future research would be needed to determine whether all event types can drive an NSW effect, or only social ones. Interestingly, some recent studies have suggested that some sub-regions in PLTC may favor the representation of social interactions over non-social ones (Wurm et al., 2017; Wurm and Caramazza, 2019; Yang et al., 2020).

### 3.5 Conclusion

The current study provides one of the first attempts to discover ERP hallmarks for the conceptual representation of events vs. coordination and agent vs. patient roles in working memory. We observed a sustained difference during a delay period for maintaining conceptual information about events relative to coordinations in working memory, which resembled the posterior-occipital negative slow wave (NSW) effect observed in previous visual working memory studies. Interestingly, a novel event role “pinging” manipulation also revealed that the NSW effect seemed to be largely dampened after the presentation of the ping.

Much future work still needs to be done to gain a better understanding of these electrophysiological hallmarks observed in our current study, such that they can be used to investigate the format of event representation in working memory. The current study is an exploratory, yet necessary first step.

## 4 Materials and methods

### 4.1 Experiment 1

#### 4.1.1 Participants

In total, 24 subjects participated in this experiment; they were balanced in terms of the list used in the main experiment. Of these subjects, data from 5 subjects were excluded: 1 of these 5 subjects was excluded because of incomplete data. Data from the other 4 of the 5 subjects were excluded based on a procedure detailed in Section 4.1.5. The remaining 19 subjects analyzed had an age of 18–27 (*M* = 22, 11 female), all right-handed as measured by a translated version of the Edinburgh Handedness Inventory (Oldfield, 1971). Subjects were recruited from the New York University Shanghai and East China Normal University community. Subjects received monetary reimbursement for their participation. Written consent was acquired from each subject, and the procedures were approved by the Institutional Review Board of the University of Maryland and New York University Shanghai.

#### 4.1.2 Stimuli

A total of 144 “event pictures” about events involving two characters were constructed, adapted from stimuli used in Hultén et al. (2014). The Hultén et al. (2014) stimuli originally consisted of cartoons depicting a single animal conducting a certain action or standing still (side-facing, used as the patients in event pictures, or front-facing, used in coordination pictures; for the definition of coordination pictures see below).

The characters used in the stimuli set were four animals (the elephant 大象, the lion 狮子, the hippo 河马 and the sheep 绵羊, therefore, there are 6 combinations). Each event involved one of 6 distinct actions (hitting 打, pulling 拉拽, kissing 亲, pointing at 指, pushing 推, scolding 骂). From each of the 6 character duos (e.g., the elephant and the lion), each of the 6 actions (e.g., hitting), and each of the 2 ways of allocating event roles (e.g., the elephant hitting the lion vs. the lion hitting the elephant), we constructed 72 unique event pictures. Note that due to the nature of the Hultén et al. (2014) stimuli (the animals performing an action were all right-facing), the agent was always to the left in these 72 “original” pictures. Therefore, we created another 72 mirror-flipped pictures by mirror-flipping the 72 original pictures to balance the relative visual position of the two characters, thus resulting in 144 event pictures in total.

Another 12 “coordination pictures” were also constructed, with two characters (also within the elephant, the lion, the hippo and the crocodile) standing next to each other. For each animal pair (X,Y), we created separate pictures in which animal X is to the left or animal Y is to the left.

The same event (e.g., lion hitting elephant) was only presented once for each subject. In order to balance relative right/left positions, we distributed the 144 original and mirror-flipped pictures across two lists (list A and B), each containing 72 pictures representing unique events; in each list, a certain character had an equal probability of appearing to the left as agent, to the left as patient, to the right as agent, and to the right as patient. Besides, for each action, the probability of the agent being to the left was the same as the probability of the agent being to the right. Both list A and list B contained an equal amount of original (i.e., 36) and mirror-flipped (i.e., 36) event pictures. Each list also included 72 coordination pictures (6 repetitions of each of the 12 coordination pictures). That is, each of the two lists consisted of 144 pictures in total (72 event pictures and 72 coordination pictures); this corresponds to 144 trials that each subject went through (72 event trials and 72 coordination trials). For more examples of our picture stimuli, see Supplementary Figure 1.

Each trial contained a single word “ping” stimulus during the delay between the picture and the probe linguistic expression. These stimuli were arranged so that within each list, (a) there was an equal probability for the agent on the left, the agent on the right, the patient on the left and the patient on the right to be pinged, (b) for each action, the agent and patient were equally likely to be pinged, (c) for each pinged character, there was an equal possibility that this character was the agent or the patient.

The format of probe linguistic expressions is illustrated in Table 1. In trials where an event picture (e.g., a picture depicting a lion hitting an elephant) had been presented (i.e., event trials), the linguistic expression (here, a sentence) either matched with the event picture (50% probability, e.g., “the lion hit the elephant”/”the elephant was hit by the lion”) or was different in event role assignment compared to the event picture (50% probability, e.g., “the elephant hits the lion”/”the lion was hit by the elephant”). The sentence was determined randomly to be in active or passive form. In trials where a coordination picture (e.g., a picture depicting a lion standing next to an elephant) had been presented (i.e., coordination trials), this linguistic expression (here, a phrase) either matched with the picture (50% probability, e.g., “the lion and the elephant”/”the elephant and the lion”) or was different in character compared to the coordination picture (50% probability, e.g., “the lion and the hippo,” “the elephant and the hippo,” etc.). The order of the two animals was determined randomly.

**TABLE 1:** Example of linguistic expressions presented in Experiment 1.

Event condition	Active expression	狮子	打了	大象	
	*Pinyin* transcription	shizi	da-le	daxiang	
	English translation	lion	hit-ASP	elephant	
		*“(the/a) lion hit (the/an) elephant”*			
	Passive expression	大象	被	狮子	打了
	*Pinyin* transcription	daxiang	bei	shizi	da-le
	English translation	elephant	BEI	lion	hit-ASP
		*“(the/an) elephant was hit by (the/a) lion”*			
Coordination condition	Coordination expression	狮子	和	大象	
	*Pinyin* transcription	shizi	he	daxiang	
	English translation	lion	and	elephant	
		*“(the/an) elephant and (the/a) lion”*			

#### 4.1.3 Procedure

Each trial started with a “ + “ in the middle of the screen (for 500 ms) signaling the beginning of a trial. This “ + “ was followed by the presentation of an event picture for 1,000 ms (for example, a picture depicting the lion hitting the elephant) or a coordination picture (for example, a picture depicting the lion standing next to the elephant). After 900 ms of a blank screen, a brief “pinging” word in larger font (250 pt) referring to one of the two characters (e.g., “lion” or “elephant”) was presented for 200 ms, in order to highlight the just-encoded character-role pairing maintained in working memory. This ping was followed by a blank screen for 600 ms, resulting in a total SOA (stimulus onset asynchrony) of 800 ms between ping presentation and the subsequent probe linguistic expression (a sentence or a phrase). After the delay period, a linguistic expression (see Table 1) appeared at the center of the screen, which either matched or did not match with the picture. The whole linguistic expression was presented on the same screen.

**FIGURE 10:**
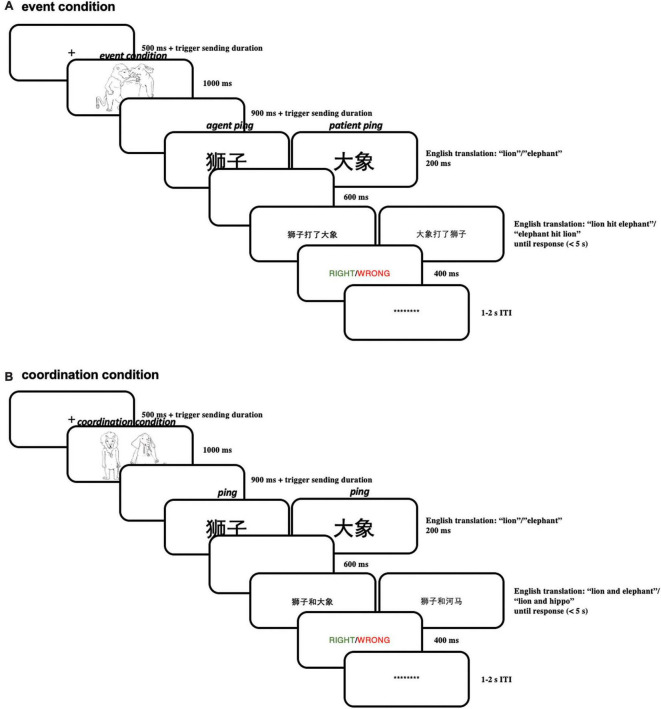
**(A)** Illustration of one trial in the event condition in Experiment 1; **(B)** illustration of one trial in the coordination condition in Experiment 1.

Subjects were asked to make a response on the keyboard whether the linguistic expression matched with the picture in the same trial as fast and accurately as possible; subjects were allocated into two response hand groups: in one group subjects pressed “z” with the left hand when matching and pressed “m” with the right hand when not; in the other group subjects pressed “m” with the right hand when matching and pressed “z” with the left hand when not. Then a feedback screen displaying whether the choice was right (in green) or wrong (in red) was presented for 400 ms; if the subjects did not make the response within 5 s, the feedback screen would automatically appear, showing “no response” (in white). After the feedback screen, there was an inter-trial interval (ITI) uniformly random across 1,000–2,000 ms before the next trial. Within the ITI, a line of asterisks appeared on the screen; before the experiment, the subjects were encouraged to blink during this period, in order to reduce blinks within trials. Subjects were instructed to always fixate on the center of the screen within a trial. For a schematic illustration of one trial in Experiment 1 see Figure 10.

Before the main experiment, subjects were given 24 practice trials (12 event pictures, 12 coordination pictures) randomly, constructed from two animals not involved in the experiment (which is, the cow 奶牛 and the crocodile 鳄鱼) and all 6 actions. Two balanced lists of event pictures (each containing 12 event pictures) were similarly created. The practice trials were used to familiarize subjects with the verbs used for the actions, as well as the response for coordination sentences, etc. These 24 practice trials were administered repetitively until the subjects reached 100% accuracy on all 24 practice trials. Note that in practice trials, the unmatching linguistic expressions for coordination pictures were constructed by substituting one of the characters (i.e., the cow or the crocodile) for another animal that was not involved in this experiment (which is, the squirrel 松鼠). This difference in the probe phrases compare to our main experiment was unlikely to affect our results, as this does not change essentially that in the coordination conditions, subjects were representing the two characters as two grouped entities.

In the main experiment, five self-paced breaks were given every 24 trials (there were in total 144 trials). The experiment was run with Psychtoolbox 3 (Brainard, 1997; Kleiner et al., 2007) in MATLAB 2020b. The background of the screen was gray, and the texts were white. The pictures were created in 1,600 × 1,200 px, and were presented on the screen as 24° × 18°. All texts, except for the ping, were presented with a font size of 100 pt; the pings were presented with a font size of 250 pt. Subjects sat at a distance of 80 cm before the screen.

#### 4.1.4 Behavioral data analysis

The reaction times were first averaged within subjects for all the correct trials, then paired *t*-tests were conducted across conditions in JASP 0.16.1 (JASP Team, 2022).

#### 4.1.5 EEG recording

EEG signal was recorded using a 32-channel active electrode system (Brain Vision actiCHamp; Brain Products) with a 1,000 Hz sampling rate in an electromagnetically shielded and sound-proof room. Electrodes were placed on an EasyCap, on which electrode holders were arranged according to the 10–20 international electrode system. The impedance of each electrode was kept below 10 kΩ. The data were referenced online to electrode Cz. Two additional EOG electrodes (HEOG and VEOG) were attached for monitoring ocular activity. The EEG data were acquired with Brain Vision PyCoder software and filtered online between DC and 200 Hz with a notch filter at 50 Hz.

#### 4.1.6 EEG data analysis

EEG data were analyzed using customized MATLAB (version: R2020b) scripts based on EEGLAB v2021.0 (Delorme and Makeig, 2004) and ERPLAB v8.20 (Lopez-Calderon and Luck, 2014). Bad channels (identified by visual inspection, 0–1 channels for each subject) were first interpolated (spherical); throughout the whole analyzing pipeline, only scalp electrodes were included. Then the EEG data were re-referenced using average (because the ICLabel plug-in was trained on average-referenced data, Pion-Tonachini et al., 2019), then down-sampled to 256 Hz, using functions in EEGLAB. Then the data were filtered by an IIR Butterworth filter with a cut-off at 0.01 Hz and 40 Hz using functions in ERPLAB (DC bias was also removed). Then ICA was conducted with the runica algorithm, and components that were labeled (using the ICLabel plug-in, Pion-Tonachini et al., 2019) with labels “Muscle,” “Eye,” “Heart,” “Line Noise,” or “Channel Noise” with a confidence of ≥ 90% were removed from data. Each subject had 0–2 components removed.

Epochs of −500:3500 ms were extracted (time locked to picture onset or ping onset), baseline corrected using the period −200 ms to 0 before picture onset. Epochs in which at least one electrode had a range of more than 100 μV during the time interval of interest (picture: −200:3500 ms, ping: −200:1800 ms) were excluded. For each subject, if there was at least one condition (i.e., event picture, coordination picture, agent ping, patient ping) that had fewer than 20 epochs, this subject was excluded. This criterion excluded the 4 subjects mentioned in Section 4.1, resulting in 19 subjects analyzed.

For the event picture vs. coordination picture comparison, we selected only −200:3500 ms epochs time-locked to picture onset that passed the above exclusion criteria (a range of no more than 100 μV) in these 19 subjects. We first averaged the ERPs within subjects across epochs for each electrode. For the NSW ROI analysis, we averaged the response in the NSW ROI (P7 and P8) for each subject, then conducted cluster-based permutation test across time (Maris and Oostenveld, 2007) by the permutest() function (Gerber, 2021) comparing the response for the NSW ROI for event picture vs. coordination picture conditions. In the cluster-based permutation tests, first, potential clusters were identified with point-to-point paired *t*-test at a threshold of alpha = 0.05 (two-tailed). Then, the t values of these potential clusters were compared against a permuted (permutations = 9,999) distribution of t values, with a threshold of alpha = 0.05 (two-tailed).

For the agent ping vs. patient ping comparison, we selected only −200:1800 ms epochs time-locked to ping onset that passed the above exclusion criteria (a range of no more than 100 μV) in these 19 subjects. We first averaged the ERPs within subjects across epochs for each electrode. For the CPP ROI analysis, we averaged the response in the CPP ROI (CP1, CP2, Pz) for each subject, then conducted cluster-based permutation test (Maris and Oostenveld, 2007) by the permutest() function (Gerber, 2021) comparing the response for the CPP ROI for agent ping vs. patient ping conditions. Because we did not observe significant clusters in this pre-determined analysis, we then exploratorily conducted a cluster-based permutation test across time with the same parameters in each of the electrodes separately.

After beginning data analysis, we discovered an inaccuracy in the timing parameters of Experiment 1 which introduced jitter between the actual presentation of the picture and the recording of the event by the EEG system. Fortunately, a photodetector signal accompanied the ping presentation, allowing a precise estimation of its actual timing. We used this validated time estimate for the ping presentation to infer the timing of the picture presentation, but this means the estimate of the picture onset timing is less direct. In the Experiment 2 replication we used a different presentation code and oscilloscope testing to ensure precise temporal alignment of the trigger codes for both picture presentation and ping presentation.

### 4.2 Experiment 2

#### 4.2.1 Participants

In total, 23 subjects participated in this experiment. Of these subjects, data from 7 subjects were excluded: 2 subjects was excluded because of incomplete data; 1 subject was excluded because of low behavioral accuracy (63%); 4 subjects were excluded based on the same procedure as Experiment 1, detailed in Section 4.1.5. The remaining 16 subjects had an age of 18–30 (*M* = 24, 8 female). 13 of them were right-handed and 3 of them were left-handed, as measured by the Edinburgh Handedness Inventory (Oldfield, 1971). They were balanced in terms of the list used in the main experiment, the list used in the practice session, and response hand. Subjects were recruited from the University of Maryland, College Park community. Subjects received monetary reimbursement for their participation. Written consent was acquired from each subject, and the procedures were approved by the Institutional Review Board of the University of Maryland.

#### 4.2.2 Stimuli

The picture stimuli in Experiment 2 were the same as Experiment 1. The probe linguistic expressions in Experiment 2 were the same, except that they were in English: e.g., “the lion pointed at the elephant,” “the sheep was hit by the lion,” “the lion and the hippo.” The pings in Experiment 2 were English words in capital letters, e.g., “LION.”

#### 4.2.3 Procedure

The procedure of Experiment 2 is generally similar to Experiment 1, except for the timings and font sizes (modified because of a smaller screen). Each trial started with a “ + “ in the middle of the screen (for 500 ms) signaling the beginning of a trial. This “ + “ was followed by the presentation of an event picture for 400 ms or a coordination picture. After 875 ms of a blank screen, a brief “pinging” word in capital letters and larger font (100 pt) referring to one of the two characters (e.g., “LION” or “ELEPHANT”) was presented for 200 ms, in order to highlight the just-encoded character-role pairing maintained in working memory. This ping was followed by a blank screen for 1,500 ms. After the delay period, a linguistic expression in English appeared at the center of the screen, which either matched or did not match with the picture. The whole linguistic expression was presented on the same screen.

**FIGURE 11:**
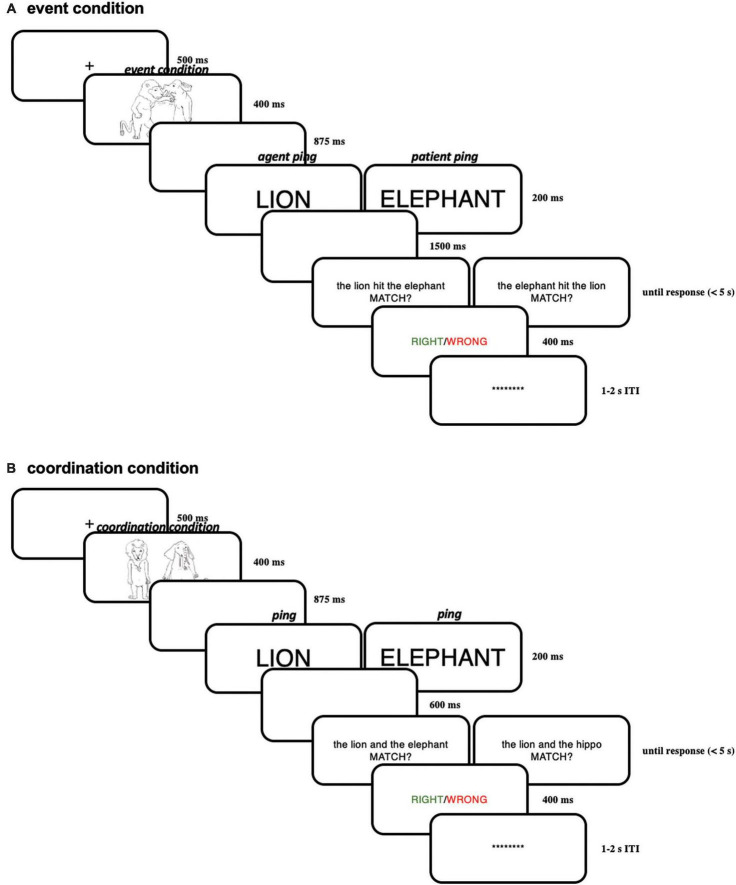
**(A)** Illustration of one trial in the event condition in Experiment 2; **(B)** illustration of one trial in the coordination condition in Experiment 2.

Subjects were asked to make a response on the keyboard whether the linguistic expression matched with the picture in the same trial as fast and accurately as possible. Then a feedback screen displaying whether the choice was right (in green) or wrong (in red) was presented for 400 ms; if the subjects did not make the response within 5 s, the feedback screen would automatically appear, showing “no response” (in white). After the feedback screen, there was an inter-trial interval (ITI) uniformly random across 1,000–2,000 ms before the next trial. Within the ITI, a line of asterisks appeared on the screen; before the experiment, the subjects were encouraged to blink during this period, in order to reduce blinks within trials. Subjects were instructed to always fixate on the center of the screen within a trial. For a schematic illustration of one trial in Experiment 2 see Figure 11.

The background of the screen was gray, and the texts were white. The pictures were presented on the screen as 24° × 18°. All texts, except for the ping, were presented with a font size of 30 pt; the pings were presented with a font size of 100 pt. The distance between the subject and the screen was measured individually.

#### 4.2.4 Behavioral data analysis

Same as Experiment 1.

#### 4.2.5 EEG recording

Twenty-nine tin electrodes were held in place on the scalp by an elastic cap (Electro-Cap International, Inc., Eaton, OH) in a 10–20 configuration. Bipolar electrodes were placed above and below the left eye and at the outer canthus of both eyes to monitor vertical and horizontal eye movements. Additional electrodes were placed over the left and right mastoids. Scalp electrodes were referenced online to the left mastoid. The ground electrode was positioned on the scalp in front of Fz. Impedances were maintained at less than 20 kΩ for all scalp electrode sites, and less than 10 kΩ for mastoid and ocular electrodes. The EEG signal was amplified by a NeuroScan SynAmps^®^ Model 5083 (NeuroScan, Inc., Charlotte, NC) with a bandpass of 0.05–100 Hz and was continuously sampled at 500 Hz by an analog-to-digital converter.

#### 4.2.6 EEG data analysis

The analysis pipeline was the same as Experiment 1 (Section 4.1.6), except that for Experiment 2, the CPP ROI was Cz, CPz and Pz. There were 0–3 interpolated bad channels for each subject, and 0–1 ICA component was removed for each subject.

## Data availability statement

The raw data supporting the conclusions of this article will be made available by the authors, without undue reservation.

## Ethics statement

The studies involving humans were approved by the Institutional Review Board of the University of Maryland and New York University Shanghai. The studies were conducted in accordance with the local legislation and institutional requirements. The participants provided their written informed consent to participate in this study.

## Author contributions

XY: Conceptualization, Data curation, Formal analysis, Investigation, Methodology, Software, Visualization, Writing—original draft, Writing—review and editing. JL: Data curation, Investigation, Software, Writing—review and editing. HZ: Data curation, Investigation, Software, Writing—review and editing. XT: Resources, Supervision, Writing—review and editing. EL: Conceptualization, Funding acquisition, Methodology, Resources, Supervision, Writing—original draft, Writing—review and editing.

## Footnotes

1 Note that there are many types of features. For example, one can make the distinction between perceptual (e.g., color, shape) and conceptual (e.g., occupation, edibility) features. It is as yet unclear what type(s) of features can be bound to object indexicals, or that all types of features can be bound to object indexicals. It is also debated whether perceptual and conceptual features are bound to the same set of object indexicals or separate sets of object indexicals (see Brody, 2020; Murez et al., 2020; Yu and Lau, 2023). In the current paper we assume that they are bound to the same set of indexicals: increasing the number of conceptual objects will elicit a similar ERP effect to increasing the number of visual objects (see Section 3.1).

2 Note that the exact duration of the NSW effect is under debate; some reports suggested that it could sustain for > 3 s (Ruchkin et al., 1992), while some other reports suggested that it terminates ∼ 1 s after memorandum onset (Fukuda et al., 2015). The duration of the CDA is unclear as well (e.g., Perez and Vogel, 2012; Li and Noguchi, 2022). Here we simply note that the NSW and the CDA have broadly similar time courses in the sense that they are both late sustaining components.

3 In contrast to these studies on visual objects, the results of Morrison et al. (2020) suggest that holding 1, 3, 5 visually-presented *digits* does not drive a sustained parietal effect, at least from visual inspection of their figures, although the electrodes selected in that study (P3, Pz, P4) were less occipital compared to other NSW studies. Digits may encourage phonological rehearsal, whose load is manifested more in frontal and/or central electrodes (Alunni-Menichini et al., 2014; Lefebvre and Jolicæur, 2016; Simal and Jolicoeur, 2020; Bidelman et al., 2021).

4 Note that there have been many fMRI studies comparing events (using pictorial or sentential stimuli) against various types of baselines (e.g., Centelles et al., 2011; Quadflieg et al., 2015; Isik et al., 2017; Okruszek et al., 2018; Matchin et al., 2019b), but due to the relatively poor temporal resolution of fMRI, it is hard to disentangle the extraction and maintenance phases, while the maintenance phase is of our current interest.

5 Note that, just as the posterior parietal indexicals for objects are not thought to represent the features of the objects themselves but to connect to those features represented elsewhere, according to this hypothesis the indexicals for event relations connect to relevant event features and knowledge elsewhere.

6 Other possibilities will be discussed in Section 3.2.

7 Notably, the reaction times seemed to be longer for Experiment 2 compared to Experiment 1, despite similar stimulus-onset asynchrony across the picture and the linguistic probe. This is in line with the observation in Zhang et al. (2021): although holding content in the activity-silent mode for a longer time does not always come with a lower behavioral accuracy, it can come with a longer reaction time to the probe.
