# Supplementary material for: Electrophysiological hallmarks for event relations and event roles in working memory
Source: Front Neurosci. 2024 Jan 24;17:1282869. doi: 10.3389/fnins.2023.1282869 (PMC10847304; doi:10.3389/fnins.2023.1282869)
Supplement: Supplementary file 1 [file Image_1.pdf]

Supplementary Materials

Electrophysiological hallmarks for event relations and event roles in working memory

Xinchi Yu[1,2], Jialu Li[3,4,5], Hao Zhu[3,4,5], Xing Tian[3,4,5], Ellen Lau[1,2]

[1] Program of Neuroscience and Cognitive Science, University of Maryland, College Park, MD, USA
[2] Department of Linguistics, University of Maryland, College Park, MD, USA
[3] Division of Arts and Sciences, New York University Shanghai, Shanghai, China
[4] Shanghai Key Laboratory of Brain Functional Genomics (Ministry of Education), School of Psychology and Cognitive Science, East China Normal University, Shanghai, China
[5] NYU-ECNU Institute of Brain and Cognitive Science at NYU Shanghai, Shanghai, China

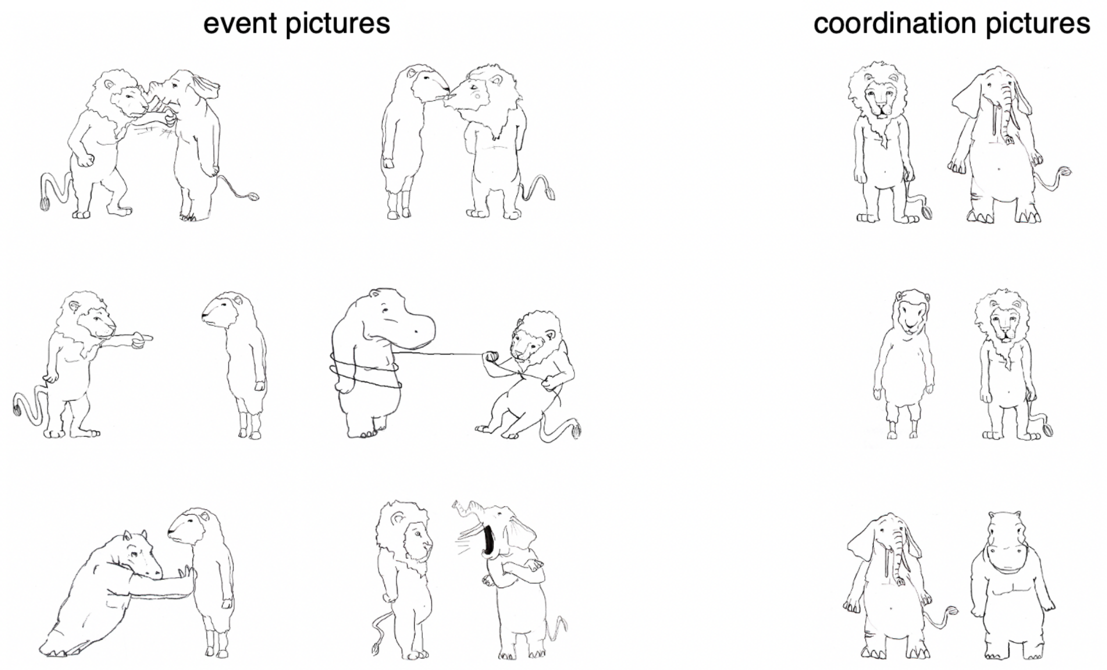

Figure S1. Examples of the event pictures and coordination pictures used in the current experiment.
